# COVID-19 vaccine effectiveness against symptomatic infection and hospitalisation in Belgium, July 2021 to May 2022

**DOI:** 10.2807/1560-7917.ES.2023.28.26.2200768

**Published:** 2023-06-29

**Authors:** Toon Braeye, Joris A F van Loenhout, Ruben Brondeel, Veerle Stouten, Pierre Hubin, Matthieu Billuart, Pui Yan Jenny Chung, Mathil Vandromme, Chloé Wyndham-Thomas, Koen Blot, Lucy Catteau

**Affiliations:** 1 Department of Epidemiology and public health, Sciensano, Brussels, Belgium

**Keywords:** COVID-19, SARS-CoV-2, vaccine, vaccine effectiveness, symptomatic infection, hospitalisation, test-negative

## Abstract

**Background:**

The Belgian COVID-19 vaccination campaign aimed to reduce disease spread and severity.

**Aim:**

We estimated SARS-CoV-2 variant-specific vaccine effectiveness against symptomatic infection (VEi) and hospitalisation (VEh), given time since vaccination and prior infection.

**Methods:**

Nationwide healthcare records from July 2021 to May 2022 on testing and vaccination were combined with a clinical hospital survey. We used a test-negative design and proportional hazard regression to estimate VEi and VEh, controlling for prior infection, time since vaccination, age, sex, residence and calendar week of sampling.

**Results:**

We included 1,932,546 symptomatic individuals, of whom 734,115 tested positive. VEi against Delta waned from an initial estimate of 80% (95% confidence interval (CI): 80–81) to 55% (95% CI: 54–55) 100–150 days after the primary vaccination course. Booster vaccination increased initial VEi to 85% (95% CI: 84–85). Against Omicron, an initial VEi of 33% (95% CI: 30–36) waned to 17% (95% CI: 15–18), while booster vaccination increased VEi to 50% (95% CI: 49–50), which waned to 20% (95% CI: 19–21) 100–150 days after vaccination. Initial VEh for booster vaccination decreased from 96% (95% CI: 95–96) against Delta to 87% (95% CI: 86–89) against Omicron. VEh against Omicron waned to 73% (95% CI: 71–75) 100–150 days after booster vaccination. While recent prior infections conferred higher protection, infections occurring before 2021 remained associated with significant risk reduction against symptomatic infection. Vaccination and prior infection outperformed vaccination or prior infection only.

**Conclusion:**

We report waning and a significant decrease in VEi and VEh from Delta to Omicron-dominant periods. Booster vaccination and prior infection attenuated these effects.

**Key public health message**

**What did you want to address in this study?**

We wanted to investigate the effectiveness of vaccines against COVID-19 by comparing the risk of developing symptomatic illness and of hospitalisation in vaccinated and unvaccinated individuals. We examined if time since vaccination and if Delta and Omicron variants impacted on vaccine effectiveness. We also looked into protection by prior infection.

**What have we learnt from this study?**

We observed less symptomatic illness and hospitalisation in vaccinated compared to unvaccinated individuals, especially in the first 50 days after vaccination. Prior infection and booster vaccination increased protection. Since the Omicron variant emerged, symptomatic illness was observed more frequently in vaccinated individuals and, while the effect was smaller, the protection by vaccines against hospitalisation also decreased.

**What are the implications of your findings for public health?**

While COVID-19 vaccines remained effective over the study period (July 2021–May 2022), the effectiveness of the vaccines decreased over time and vaccines were less effective against the Omicron variant. Booster vaccination increases protection against illness and hospitalisation, even in previously infected individuals. We should keep monitoring vaccine effectiveness and consider timely booster-campaigns.

## Introduction

Belgium started the roll-out of its COVID-19 vaccination campaign on 28 December 2020, initially targeting nursing homes residents and personnel. When a period of high COVID-19 incidence occurred in March and April 2021 associated with the severe acute respiratory syndrome coronavirus 2 (SARS-CoV-2) Alpha variant of concern (VOC) (Phylogenetic Assignment of Named Global Outbreak (Pango) lineage designation B.1.1.7), around 90% of nursing home residents and around 37% of healthcare professionals had completed their primary vaccination schedule [1,2]. Vaccination coverage in the adult population (aged ≥ 18 years) increased to over 80% by October 2021. Despite this high coverage and the start of a booster campaign in September 2021, the Delta VOC (Pango lineage designation B.1.617.2) resulted in considerable intensive care unit (ICU) and hospital occupancy from October to December 2021. The Delta variant showed immune escape which, in combination with waning of vaccine-induced protection, lowered vaccine effectiveness (VE) [3]. 

In an effort to increase the nationwide vaccine-induced protection against SARS-CoV-2, the booster campaign was accelerated and vaccination centres were scaled up. The relevance of the booster campaign further increased when the Omicron VOC (Pango lineage BA.1.1.529) became dominant in January 2022, as a booster dose was required to effectively neutralise Omicron [4]. On 28 February 2022, 88.9% of all Belgians aged 18 years and above had completed primary vaccination and 74.4% had received a booster dose. The cumulative Belgian incidence of laboratory-confirmed SARS-CoV-2 infections at that time was 3.57 million over 11.6 million residents.

Observational [5-9] and in vitro studies [10,11] have reported that the effectiveness of COVID-19 vaccines decreases over time since vaccination, a process typically referred to as waning, and that several VOCs have shown some degree of immune escape from both vaccine-induced and infection-induced immunity. The Omicron variant has been especially associated with an increased risk of breakthrough infection [12,13]. Quantifying these temporal and variant-specific vaccination effects as well as the confounding effect of infection-acquired immunity is necessary to obtain timely and detailed VE estimates. These estimates are essential to continue the development of the vaccination strategy.

In the present study, we estimated VE against symptomatic infection (VEi) and hospitalisation (VEh) associated with primary and booster vaccination from Belgian healthcare databases over the second half of 2021 and the first half of 2022, while adjusting for age, sex, residence, dominant VOC, prior infection and time since vaccination. In addition, we estimated protection offered by prior infection and hybrid immunity, i.e. immunity resulting from the combination of SARS-CoV-2 infection and vaccination.

## Methods

### Datasets and study period

This study was part of the LINK-VACC (LINKing of registers for COVID-19 VACCine surveillance) project. This project allowed the linkage of selected variables from multiple existing national health and social sector registries on the individual level using the Belgian social security identification number within a pseudonymised environment. Three COVID-19 registries were used for the present study: the vaccination registry, the laboratory testing registry and the clinical hospital survey. The vaccination and laboratory testing registries are nationwide and exhaustive. The clinical hospital survey is a voluntary survey. The participating hospitals reported 45% (20,250/45,312) of all hospital admissions for COVID-19 in Belgium during the study period (12 July 2021–26 May 2022).

Since relatively few samples from individuals with SARS-CoV-2 infections were sequenced (5.4% of laboratory-confirmed infections during the study period), the period during which the Delta or Omicron VOC was detected in at least 80% of the samples sequenced in Belgium’s baseline genomic surveillance was used as a proxy for Delta and Omicron infections [14]. Based on this surveillance, the Delta period began on 12 July 2021 (start of the study period) and ended 22 December 2021 and the Omicron period included in this study started 4 January 2022 and concluded 26 May 2022 (end of the study period). We did not differentiate between the different Omicron sub-lineages BA.1 and BA.2. The period of BA.1 dominance was short (around 1 month, January 2022) and followed by BA.2 dominance.

### Immunity status: vaccination and prior infection

Immunity status was defined by: (i) the vaccination status (unvaccinated, primary or booster vaccination), (ii) time since last vaccination in 50-day blocks (from the date of last administered vaccine) and (iii) most recent prior infection. Prior infections were defined as a laboratory-confirmed SARS-CoV-2 infection (antigen or PCR test) and categorised into four periods by time of sampling: 2020 (Jul–Dec), first half of 2021 (Jan–Jun), second half of 2021 (Jul–Dec) and since 2022 (Jan–Mar). Positive tests within 60 days of a previous positive test were discarded as prior infection as they possibly diagnosed the same infection.

Belgium predominantly used two viral vector vaccines (Vaxzevria (ChAdOx1, nCoV-19, Oxford-AstraZeneca) and Janssen vaccine (Ad26.COV2-S, Janssen-Cilag International NV) for primary vaccination and two mRNA vaccines (Comirnaty (BNT162b2, BioNTech-Pfizer) and Spikevax (mRNA-1273, Moderna)) for both primary and booster vaccination. We defined a complete primary vaccination schedule as having received two doses of vaccine, except for Janssen vaccine, for which one dose sufficed. Booster vaccination was defined as vaccination with an mRNA vaccine after completion of the primary vaccination schedule. In mid-December 2021, the interval between primary and booster vaccination for mRNA vaccines was shortened from 6 to 4 months; the interval between a viral vector vaccine-based priming and a booster shot remained at 2 months for Janssen vaccine (Ad26.COV2-S) and 4 months for ChAdOx1). Individuals partially vaccinated, i.e. one dose of a two-dose schedule, or vaccinated with a mixed primary vaccination schedule were excluded. Vaxzevria and Spikevax vaccines were considered effective 14 days, Comirnaty 7 days and Janssen 21 days after administration. Booster doses were considered effective 7 days after administration. Samples taken in the period between vaccine administration and assumed effectiveness were excluded from the analysis. 

### Test-negative design

The study population consisted of all Belgian adults (≥ 18 years old) who self-reported COVID-19-symptoms and were tested for SARS-CoV-2 by PCR (with a date of sampling 3 days before to 7 days after symptom onset). We included individuals only once in the analysis to avoid an effect of individual-specific healthcare-seeking behaviour. If individuals reported multiple symptom onset dates, we randomly chose one per VOC period. PCR tests were free of charge and the default test method when symptomatic during the study period. In addition, rapid antigen tests were used as self-tests from December 2021 onwards. We excluded PCR tests that were performed for the confirmation of a positive self-test, since the result of a self-test possibly already depends on the individual’s vaccination status. A sensitivity analysis in which we repeat the analysis without excluding these tests can be found in Supplementary Materials S1.

Using a test-negative design, immunity status was compared between cases and controls. Cases were defined as individuals with at least one positive PCR test for SARS-CoV-2. Controls were defined as individuals with negative results for all SARS-CoV-2 PCR tests. Cases and controls were matched on age group (per 5 years), sex, province of residence (a map with Belgian provinces is provided in Supplementary Figure S4) and calendar week of sampling. Data on test results were fitted using conditional logistic regression to obtain immune status-specific adjusted odds ratios (aOR). Unvaccinated individuals without a prior infection were set as reference category. The VEi was defined as (1 − aOR) x 100.

### Proportional hazard regression

We performed a follow-up time-to-event analysis on the cases of the test-negative design (symptomatic individuals with a positive PCR test). Events were defined as hospital admissions linked to COVID-19 symptoms within 4 weeks following the positive PCR test. Individuals were censored from follow-up if they died or received a vaccine. We obtained hazard ratios (HR) through a proportional hazard (PH) regression and estimated VE against hospitalisation given symptomatic infection (VEh|i) as (1 − HR) x 100. We adjusted for age group (per 5 years), sex and province of residence.

We explored the proportional hazards assumption in Supplementary Material S2 by plotting the Kaplan-Meier curve and the survival probability obtained from the PH regression and by exploring the Schoenfeld-residuals. Since only a sample of person-level hospitalisation data were collected, the assumption that the HR based on a hospital survey is an unbiased estimator of the corresponding HR in Belgian’s hospitalised population is crucial. In Supplementary Material S3, we look into the representativeness of the sample. We found no evidence that either of the assumptions were violated. Because of small numbers, we reported only descriptive statistics for hospitalisation of individuals with a prior infection.

The VEh was obtained by combining VEi and VEh|i

VEh = 1 – (1 – VEi)*(1 - VEh|i)

in which the VE estimates are included as decimals.

### Model fit and stratification

Models were fitted on datasets stratified by VOC period. When we report VE estimates for an age group (˃ 65 years), the dataset used for coefficient estimation was limited to individuals in that age group, but within the model we adjusted for the 5-year age groups as previously mentioned. Confidence intervals (CI) were set at 95%. The R 4.1.2 and the R-package ‘survival’ were used for both the test-negative and the PH regression [15,16]. To improve the readability of the graphs and to avoid overinterpretation, we did not plot estimates for which the uncertainty was large (95% CI > 50%).

## Results

The number of PCR tests performed during the study period in symptomatic individuals was 2,608,952, including 1,254,572 during the Delta-dominant period and 1,354,380 during the Omicron-dominant period. These tests were linked to 2,126,625 individuals, 1,008,600 during the Delta-dominant period and 1,125,497 during the Omicron-dominant period. Multiple symptom-onset dates within the same VOC period were reported by 11% of individuals (233,928/2,126,625).

Tests were excluded because of incomplete primary vaccination (n = 44,981), non-standard/mixed primary vaccination (n = 9,418), PCR test for the confirmation of a positive self-test (n = 105,894), sampling in between vaccine administration and assumed effectiveness (n = 66,986), with multiple reasons possible.

A total of 1,932,546 individuals were included in the VEi analysis (Table 1). For analysis of VEh|i, 734,115 individuals were included (Table 2).

**Table 1:** Number of individuals included in the test-negative study for analysis of vaccine effectiveness against symptomatic SARS-CoV-2 infection, Belgium, 12 July 2021–26 May 2022 (n = 1,932,546)

Variant of concern	Age group (years)	Sex	Unvaccinated		Primary vaccination		Booster vaccination	
			n	Proportion positive	n	Proportion positive	n	Proportion positive
Delta	18–64	F	84,201	0.36	370,093	0.19	9,231	0.10
		M	71,652	0.37	284,582	0.20	4,286	0.12
	65–79	F	2,802	0.46	37,377	0.25	6,374	0.11
		M	1,848	0.50	28,578	0.31	5,527	0.14
	≥ 80	F	761	0.41	8,074	0.22	5,855	0.10
		M	310	0.43	5,146	0.29	3,638	0.15
Omicron	18–64	F	76,638	0.61	138,470	0.55	270,570	0.41
		M	64,407	0.61	112,776	0.55	193,621	0.40
	65–79	F	3,197	0.63	4,377	0.51	49,141	0.45
		M	1,902	0.65	2,736	0.55	38,683	0.48
	≥ 80	F	897	0.57	1,364	0.48	25,632	0.46
		M	469	0.59	771	0.51	16,560	0.53

SARS-CoV-2: severe acute respiratory syndrome coronavirus 2.

**Table 2:** Number of individuals with symptomatic SARS-CoV-2 infections included in the proportional hazard regression for analysis of vaccine effectiveness against hospitalisation, Belgium, 12 July 2021–26 May 2022 (n = 734,115)

Variant of concern	Age group (years)	Sex	Unvaccinated		Primary vaccination		Booster vaccination	
			n	Proportion in CHS	n	Proportion in CHS	n	Proportion in CHS
Delta	18–64	F	32,076	0.031	73,217	0.006	990	0.046
		M	28,331	0.049	60,362	0.011	560	0.096
	65–79	F	1,517	0.242	9,908	0.056	771	0.125
		M	1,166	0.317	9,612	0.106	901	0.178
	≥ 80	F	475	0.480	2,121	0.213	697	0.172
		M	246	0.561	1,897	0.308	657	0.253
Omicron	18–64	F	48,370	0.004	80,411	0.003	118,584	0.003
		M	40,347	0.006	64,458	0.004	80,106	0.004
	65–79	F	2,167	0.072	2,405	0.059	23,240	0.024
		M	1,395	0.151	1,658	0.101	19,591	0.043
	≥ 80	F	718	0.306	794	0.188	13,235	0.100
		M	417	0.396	519	0.268	10,196	0.146

CHS: clinical hospital survey; SARS-CoV-2: severe acute respiratory syndrome coronavirus 2.

### Vaccine effectiveness

**Figure 1:**
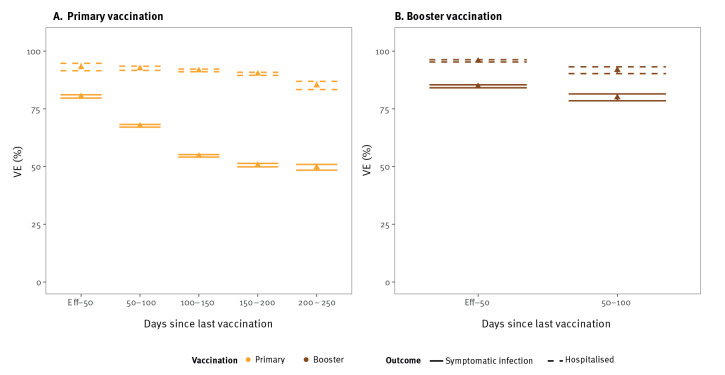
Vaccine effectiveness against symptomatic infection (n = 930,335) and hospitalisation (n = 225,504) during SARS-CoV-2 Delta dominance, Belgium, 12 July 2021–22 December 2021

**Figure 2:**
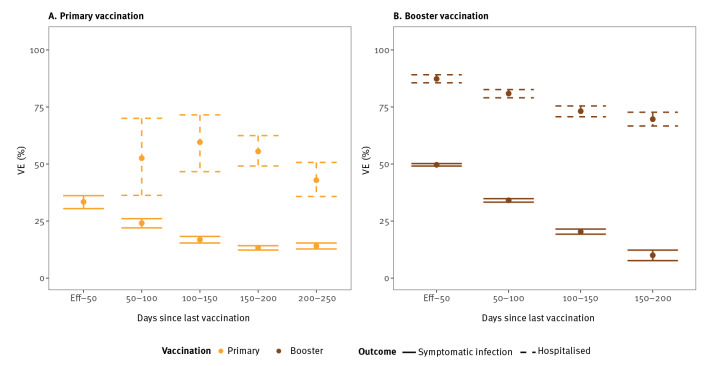
Vaccine effectiveness against symptomatic infection (n = 1,002,211) and hospitalisation (n = 508,611) during SARS-CoV-2 Omicron dominance, Belgium, 4 January–26 May 2022

The Omicron variant was associated with reduced VEi and VEh estimates compared with the Delta variant. Initially, from the point at which the vaccine was considered effective until 50 days after vaccination, Delta VEi was 80% (95% CI: 80–81) and Omicron VEi was 33% (95% CI: 30–36) for primary vaccination (Figure 1A and 2A). The initial VEi conferred by booster vaccination was 85% (95% CI: 84–85) against Delta and 50% (95% CI: 49–50) against Omicron (Figure 1B and 2B).

Eff: number of days before the vaccination is considered effective; SARS-CoV-2: severe acute respiratory syndrome coronavirus 2; VE: vaccine effectiveness.

Vaxzevria and Spikevax vaccines were considered effective 14 days, Comirnaty 7 days and Janssen 21 days after administration. Booster doses were considered effective 7 days after administration.

Number of cases presented are as follows for (A): VEi (n, vaccinated = 733,850; n, unvaccinated = 161,574), VEh (n, vaccinated = 157,117; n, unvaccinated = 63,811) and (B) VEi (n, vaccinated = 34,911; n, unvaccinated = 161,574), VEh (n, vaccinated = 4,576; n, unvaccinated = 63,811).

Eff: Number of days before the vaccination is considered effective; SARS-CoV-2: severe acute respiratory syndrome coronavirus 2; VE: vaccine effectiveness.

Vaxzevria and Spikevax vaccines were considered effective 14 days, Comirnaty 7 days and Janssen 21 days after administration. Booster doses were considered effective 7 days after administration.

Case numbers presented are as follows for (A): VEi (n, vaccinated =  260,494; n, unvaccinated = 147,510), VEh (n, vaccinated = 150,245; n, unvaccinated = 93,414) and (B) VEi (n, vaccinated = 594,207; n, unvaccinated = 147,510), VEh (n, vaccinated = 264,952; n, unvaccinated = 93,414).Infection-acquired and hybrid immunity against the Omicron variant

The VEi for primary vaccination decreased by 25 (Delta VEi) and by 16 (Omicron VEi) percentage points over a 100–150-day period, corresponding to a Delta VEi of 55% (95% CI: 54–55) and an Omicron VEi of 17% (95% CI: 15–18). Given the limited time between booster vaccination and the end of the Delta-dominant period, we could not accurately assess waning of booster vaccination against the Delta variant (Figure 1B). We observed waning of VEi against the Omicron variant; Omicron VEi was estimated at 20% (95% CI: 19–21), 100–150 days after booster vaccination, corresponding to a decrease of 30 percentage points from the initial booster Omicron VEi.

The VEh was higher than VEi. Initial Delta VEh was 93% (95% CI: 92–95) for primary vaccination and 96% (95% CI: 95–96) for booster vaccination. Against the Omicron variant, initial VEh for primary vaccination could only be estimated with large uncertainty (53%, 95% CI: 29–78). Since the groups at high-risk of hospitalisation were booster-vaccinated first, only few hospitalisations were registered among individuals with a primary vaccination during the period of Omicron dominance. For booster vaccination, we estimated initial Omicron VEh at 87% (95% CI: 86–89). We observed a decrease of Omicron VEh to 73% (95% CI: 71–75), 100–150 days after booster vaccination.

Protection against symptomatic Omicron infections, conferred by a prior SARS-CoV-2 infection in unvaccinated individuals, was higher for more recent infections compared with older infections. For prior infections from 2022, we estimated protection ((1 − aOR) x 100) at 75% (95% CI: 72–78), for infections from the second half of 2021 at 39% (95% CI: 37–40), for infections from the first half of 2021 at 23% (95% CI: 21–26) and for infections from 2020 at 14% (95% CI: 12–17).

**Figure 3:**
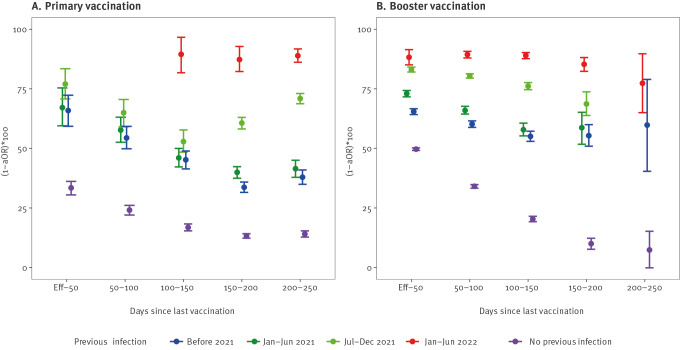
Hybrid immunity against symptomatic infection by period of prior infection and days since vaccination during SARS-CoV-2 Omicron dominance, Belgium, 4 January 2022–26 May 2022 (n = 1,002,211)

Hybrid immunity offered more protection if the last antigen exposure (either vaccination or infection) was more recent: initial hybrid protection against symptomatic infection with a prior infection in 2022 was estimated at 88% (95% CI: 85–91) and 83% (95% CI: 82–84) with a prior infection from the second half of 2021. Hybrid immunity waned at a rate comparable to primary vaccination for prior infections occurring before the second half of 2021 and slower for more recent infections. The combination of booster vaccination with prior infection continued to offer more protection than booster vaccination without prior infection, even if the prior infection was from 2020 (estimate of 65% (95% CI: 64–67) vs 50% (95% CI: 49–50) without prior infection; Figure 3).

aOR: adjusted odds ratio; Eff: Number of days before the vaccination is considered effective; SARS-CoV-2: severe acute respiratory syndrome coronavirus 2.

Vaxzevria and Spikevax vaccines were considered effective 14 days, Comirnaty 7 days and Janssen 21 days after administration. Booster doses were considered effective 7 days after administration.

Among hospitalised patients, 436 (2.9%) had a prior infection. For 223 individuals, this was an infection from 2020.

### Sex and age differences

**Figure 4:**
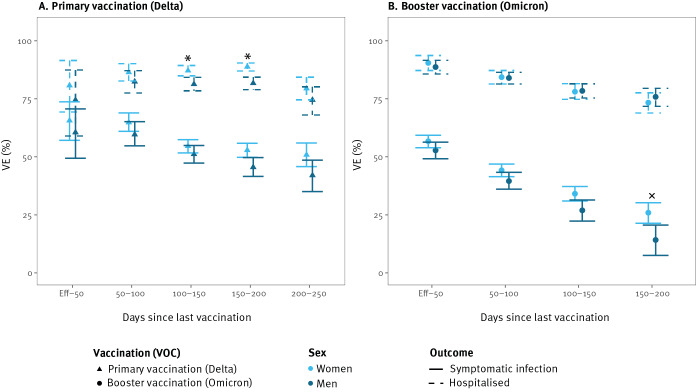
Vaccine effectiveness against symptomatic SARS-CoV-2 infection (n = 252,019) and hospitalisation (n = 106,303) in individuals of 65 years and over, Belgium, 12 July 2021–26 May 2022

Overall 95% CI of the VE estimates by sex overlapped. However, in individuals aged 65 years and over infected with the Delta variant 100–200 days after primary vaccination, we observed a statistically significant difference in VEh by sex. VEh was lower in men (150–200 days after primary vaccination) and it was estimated at 82% (95% CI: 79–84) compared with 89% (95% CI: 87–90) for women. After booster vaccination, VEi against Omicron infections seemed to wane faster for men (Figure 4). No sex-specific significant differences were found in younger age groups (< 65 years).

Eff: Number of days before the vaccination is considered effective; SARS-CoV-2: severe acute respiratory syndrome coronavirus 2; VE: vaccine effectiveness; VOC: variant of concern.

Vaxzevria and Spikevax vaccines were considered effective 14 days, Comirnaty 7 days and Janssen 21 days after administration. Booster doses were considered effective 7 days after administration.

The asterisk indicates that the 95% confidence intervals of women and men are not overlapping.

**Figure 5:**
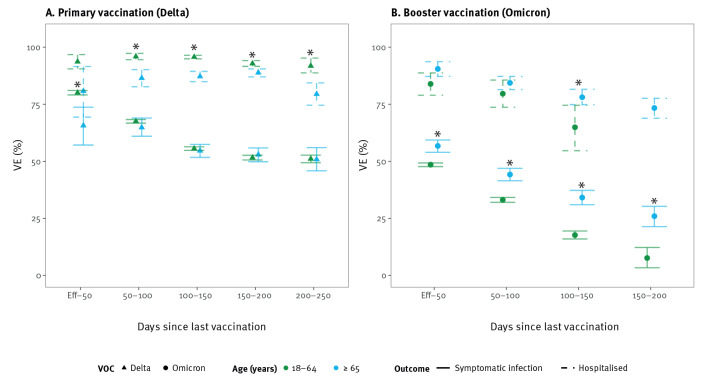
Vaccine effectiveness against symptomatic SARS-CoV-2 infection (n = 1,095,054) and hospitalisation (n = 411,696) for women aged 18–64 years and 65 years and over, Belgium, 12 July 2021–26 May

Against the Delta variant, VEh was significantly higher in younger (< 65 years) compared with older (≥ 65 years) women. The difference was inverse for VEi after booster vaccination against the Omicron variant (Figure 5), as we observed significantly higher protection in older age groups.

Eff: Number of days before the vaccination is considered effective; SARS-CoV-2: severe acute respiratory syndrome coronavirus 2; VE: vaccine effectiveness; VOC: variant of concern.

Vaxzevria and Spikevax vaccines were considered effective 14 days, Comirnaty 7 days and Janssen 21 days after administration. Booster doses were considered effective 7 days after administration.

The asterisk indicates that the 95% confidence intervals of women aged 18-64 and ≥ 65 years are not overlapping.

## Discussion

In this study, we presented the effect of vaccination and prior infection on the risk of SARS-CoV-2 VOC-specific symptomatic infection and hospitalisation in Belgium from July 2021 to May 2022. Our main findings include: (i) Omicron dominance and waning reduced VEi and VEh estimates, (ii) booster vaccination increased protection by restoring waning immunity and attenuating Omicron’s immune escape, (iii) hybrid immunity was associated with higher protection than vaccine-induced or infection-acquired immunity only and (iv) while more recent infections and vaccinations were associated with higher protection against symptomatic infection, protection from the oldest infections (over 1 year ago) and oldest vaccinations (200–250 days ago), within this study, remained significant.

Among the currently known VOCs, the Omicron variant displayed the most pronounced humoral immune escape [17]. We estimated the initial primary vaccination VE against symptomatic Omicron infections at 33% (95% CI: 30–36). However, when the Omicron variant became dominant at the start of 2022, Belgian adults without booster vaccination had lower protection (VEi of 17% (95% CI: 15–18)) because, on average, they completed their primary vaccination more than 120 days prior. Other studies have reported an initial Omicron VEi of around 36–56% [6,18] and little to no protection after  180 days [9,19,20]. The Omicron variant has also been associated with lower vaccine-induced protection against hospitalisation [19,21,22]. We estimated Omicron VEh at 56% (95% CI: 49–62), significantly lower than the 90% (95% CI: 90–91) VEh estimate against the Delta variant (both 100–150 days after primary vaccination). Booster vaccination restored waned protection and improved initial VEi estimates against both VOCs. Our findings in older age groups (≥ 65 years) of an initial Omicron VEi of 60–75% after booster vaccination is in accordance with in vitro studies [23] and epidemiological studies [6,7,18,24]. From a public health perspective, it is important to state that, while the responses of the different immunity layers are VOC-specific [25], transmission and probability of severe outcomes are also VOC-specific. The public health impact of the lower VEh should be evaluated together with the reported lower severity in most age groups and higher transmissibility of the Omicron compared with the Delta variant [9,22,26,27].

The waning of primary vaccination during the Delta-dominant period in our study is comparable to the estimates reported by a systematic review: 20–30% for VEi and 9–10% for VEh [28]. As reported by other studies, we observed slower waning of VEh compared with VEi [19,29-33]. The rate of waning of VEh after a third vaccine dose differs between studies, with estimates between 5.3% (95% CI: 2.4–8.7) [19] and 30% [34,35] over a 4-month period. Towards the end of our follow-up time, waning of VEh seemed to decelerate, while VEi estimates continued to decline. The available serological evidence describes stabilising antibody titres 6 to 9 months post vaccination [11,36]. Future research should investigate waning over longer time periods.

In accordance with immunological [11,37,38] and epidemiological [20,39-41] studies, we observed significant protection from prior infections both in vaccinated and unvaccinated individuals. In unvaccinated individuals, protection against symptomatic infection remained for over 1 year, which was the maximum follow-up timeframe included in this study. The more recent prior infections, however, conferred higher protection. This likely indicates both waning of immunity and differences in cross-neutralisation between VOCs [11,42,43]. Given the functional relevance of immunological processes such as cross-neutralisation and affinity maturation [17,44], observed time trends in convalescent individuals are specific to the sequence of VOCs. Our results might therefore be most relevant to populations that also experienced an Alpha–Delta–Omicron sequence.

In contrast to Altarawneh et al. [20], we did not observe that the effect of hybrid immunity originated solely from prior infection, as hybrid immunity was estimated as more effective. That additional protection remained after booster vaccination has illustrated the benefit of a fourth, heterologous, antigen exposure [11,36]. In contrast, studies have shown that repeated homologous antigen exposures might achieve maximal immunogenicity after three exposures [45,46]. This has a number of public health implications such as the usefulness of booster vaccination irrespective of prior infection [47] and the need for an updated/heterologous booster vaccine.

We have previously identified men over 65 years of age as the population with the lowest VE against Delta infections over time as compared to women and younger men [48]. In this study, male sex was associated with a lower VEh estimate for primary vaccination during Delta variant dominance and faster waning of VEi after booster vaccination. Our results with respect to age are less consistent. While younger individuals, under 65 years of age, were associated with higher VE estimates during Delta dominance, the association inversed during Omicron dominance. Lower VE estimates for younger age groups against Omicron have also been reported for the United States [35] and Denmark [32]. While this observation can be linked to age-specific characteristics of the Omicron variant or age-specific behavioural differences, a rise in undiagnosed infections in younger populations is another possible explanation.

This study has several limitations. With respect to the investigated outcomes, symptomatic infection is based on a self-reported date of symptom onset without further information on the actual clinical presentation. With respect to hospitalisation as an outcome, no exhaustive patient registry was available for COVID-19-related hospitalisations in Belgium. For the data used in our study, we found no arguments for a sampling bias by vaccination status. However, a missing data and measurement error problem remain. VEh estimates should be compared with care between countries as countries’ hospital admission policies differ. While the included hospitalisations were associated with COVID-19 symptoms, we did not differentiate by severity or hospital outcome.

For prior infections, we did not make the distinction between symptomatic and asymptomatic infections, while there is evidence that the protection differs by presentation [38]. We also did not differentiate between infections occurring before or after vaccination. Both have been reported to boost the quantity, quality and breadth of the humoral immune response [49,50]. We did differentiate prior infections by the time of their occurrence, but the periods we used were large and infection were not distributed uniformly over these periods. We could only include those prior infections that had been laboratory-confirmed. Given that case-ascertainment, and therefore detection of prior infections, might be age- and sex-specific, comparison of VE’s by age and sex should be done with care.

Differences in exposure by immunity status (because of heterogeneity in contacts and behaviour) remain a potential source of bias. To some extent, the required use of a certificate, e.g. either of vaccination, negative test or recovery, during the study period to enter bars, restaurants or sport and cultural facilities might have caused additional exposure differences between vaccinated and unvaccinated individuals. Future studies might opt for a reference category other than unvaccinated individuals, but since this was the first Belgian study to estimate VEh, we chose a reference group (unvaccinated individuals) that allowed for an intuitive, absolute interpretation. Using a test negative case–control design helped to control for differences in healthcare-seeking behaviour between vaccinated and unvaccinated individuals. At the time of writing, other variables that might affect exposure and testing behaviour including socioeconomic status, profession and variables on individual susceptibility such as comorbidities, prior influenza vaccination, or immune status were still being explored or not available for linkage.

The sequential roll-out of the Belgian vaccination campaign has important implications for our study. Since we did not want to make any modelling assumptions, e.g. temporal dynamics of waning, the analyses are limited by the numbers available for each discrete time period. Estimates of Delta VE for booster vaccination were driven by effectiveness in older age groups and vice versa for primary vaccination and Omicron. Consequences were (i) some estimates could not be provided and the uncertainty around others was large, (ii) estimates for all Belgian adults might be driven by certain age groups and (iii) when comparing VE estimates by time since vaccination for different age groups, we are also comparing different calendar periods. With respect to this aspect, we did not differentiate between BA.1 and BA.2 dominant periods in the analysis. While comparable immunogenicity against Omicron sublineages (BA.1, BA.2) has been reported [10,51,52], this remains one of this study’s limitations. Possible conclusions on the rate of waning might therefore be confounded by differences in immune escape between BA.1 and BA.2.

To limit the complexity of the study and increase its power, we did not differentiate between vaccine brands. Exploratory analyses were not pointing towards large differences between brands after booster vaccination. For the primary schedule, we previously explored differences between brands [48]. In addition, we excluded minors (aged ≤ 17 years) from the analysis since they were not targeted during the initial vaccination campaign.

## Conclusion

While protection remained over the different VOCs, VOC-specific immune escape and waning of vaccine-induced and infection-acquired immunity were major drivers of trends in 2021–22. Our findings quantified these effects, which can help communication on past vaccination campaign while also optimizing benefits of future vaccination campaigns. The waning rate differed between VEi and VEh. More frequent vaccination campaigns will be necessary if the objective is avoiding symptomatic infection as compared with hospitalisation. Trends in population immunity will, however, increasingly be determined by hybrid immunity which appears to wane more slowly. If some of the observed protection from hybrid immunity resulted from heterologous antigen exposures, multivalent vaccines should be considered. Finally, vaccination can be offered regardless of prior infection, as there is additional benefit either way.

## Supplementary Data

760000 Supplement
