# Supplementary material for: COVID-19 vaccine effectiveness against symptomatic infection and hospitalisation in Belgium, July 2021 to May 2022
Source: Euro Surveill. 2023 Jun 29;28(26):2200768. doi: 10.2807/1560-7917.ES.2023.28.26.2200768 (PMC10311948; doi:10.2807/1560-7917.ES.2023.28.26.2200768)
Supplement: Supplement [file 22-00768_BRAEYE_SUPPLEMENT.pdf]

COVID-19 VACCINE EFFECTIVENESS AGAINST SYMPTOMATIC INFECTION AND HOSPITALISATION IN BELGIUM, JULY 2021 TO MAY 2022: SUPPLEMENTARY MATERIALS

This supplementary material is hosted by Eurosurveillance as supporting information alongside the article “COVID-19 vaccine effectiveness against symptomatic infection and hospitalisation in Belgium, July 2021 to May 2022”, on behalf of the authors, who remain responsible for the accuracy and appropriateness of the content. The same standards for ethics, copyright, attributions and permissions as for the article apply. Supplements are not edited by Eurosurveillance and the journal is not responsible for the maintenance of any links or email addresses provided therein.

CONTENTS

S1. Including PCR-tests for the confirmation of positive self-tests: sensitivity analysis. 1
S2. Proportional hazard assumption 2
S3. Is the VE estimated from the Clinical Hospital survey an unbiased estimator of Belgium’s VE against hospitalization..4

S1. Including PCR-tests for the confirmation of positive self-tests: sensitivity analysis.

PCR-tests that were performed for the confirmation of a positive self-test were excluded from the main analysis. We argued that the result of the self-test was already possibly partly defined by vaccine effectiveness. The vaccine effectiveness within this population therefore has a different interpretation (not absolute, but conditional given a positive self-test) compared to an untested population. To further explore this, and a possible selection bias, we conducted a sensitivity analysis. We included the PCR-tests performed for the confirmation of a positive self-test in the analysis. We present the corresponding figure 1 and 2 from the main analysis (Supplementary figures 1 and 2).
When compared to the main analysis, the estimates of the sensitivity analysis are slightly lower.

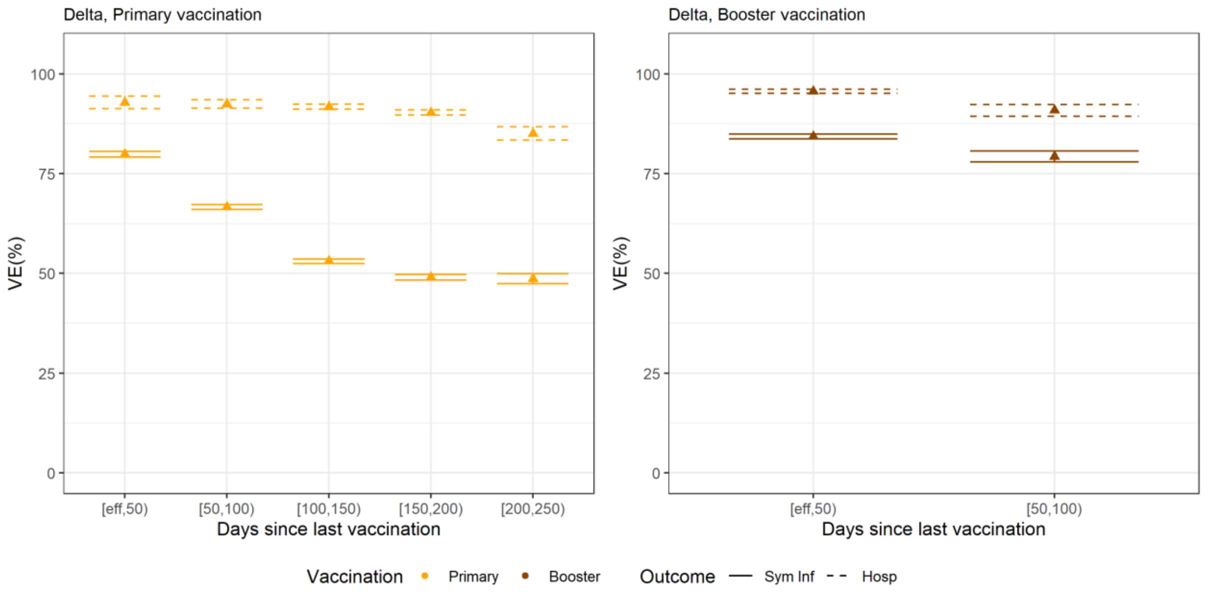

Supplementary Figure 1: Vaccine Effectiveness against symptomatic infection (Sym Inf) and hospitalization (Hosp), (left) primary-vaccination, (right) booster-vaccination, adults, both sexes, Delta, 12/07/2022 – 22/12/2021, Belgium. Also including PCR-tests performed for the confirmation of a positive self-test.

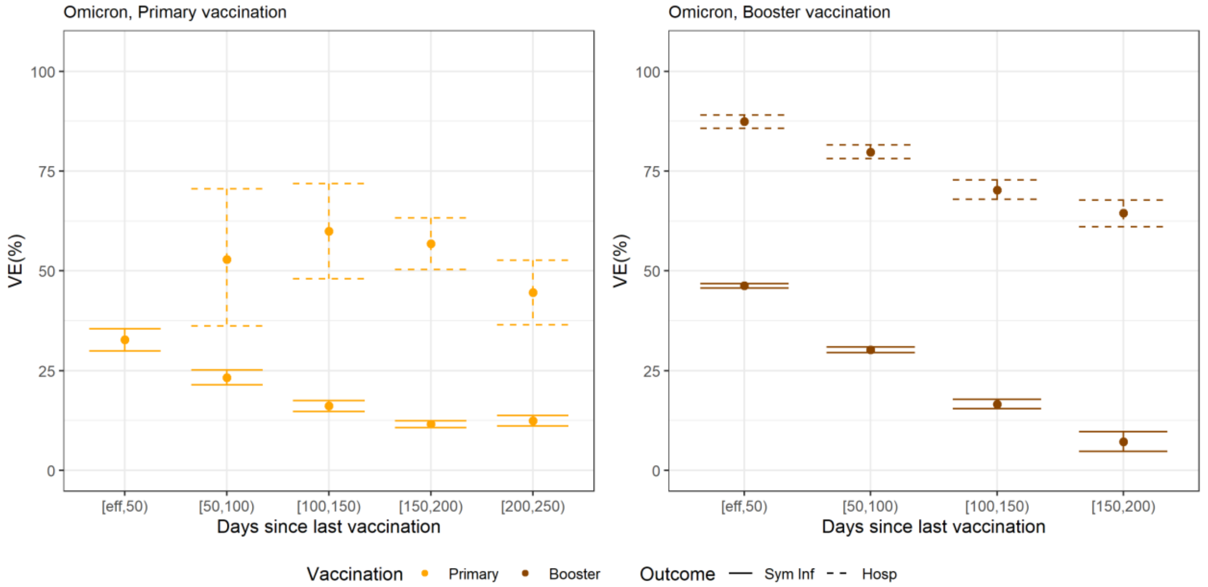

Supplementary Figure 2: Vaccine Effectiveness against symptomatic infection (Sym Inf) and hospitalization (Hosp), (left) primary-vaccination, (right) booster-vaccination, adults, both sexes, Omicron, 04/01/2022 – 26/05/2022, Belgium. Also including PCR-tests performed for the confirmation of a positive self-test.

S2. Proportional hazard assumption

We first illustrate the probability of survival (no hospitalization linked to Covid-19 reported to the clinical hospital survey) over time by (1) the Kaplan-Meier estimator by vaccination status and (2) the adjusted proportional hazard (Cox-regression) model.

The Kaplan-Meier curve is not adjusted for age group or time since vaccination, we limited the data used for the fit to those aged 65 years and over and the initial effect of vaccination [eff,50 days) for samples taken during the period of Delta-dominance in persons without prior infection (Supplementary Figure 3).

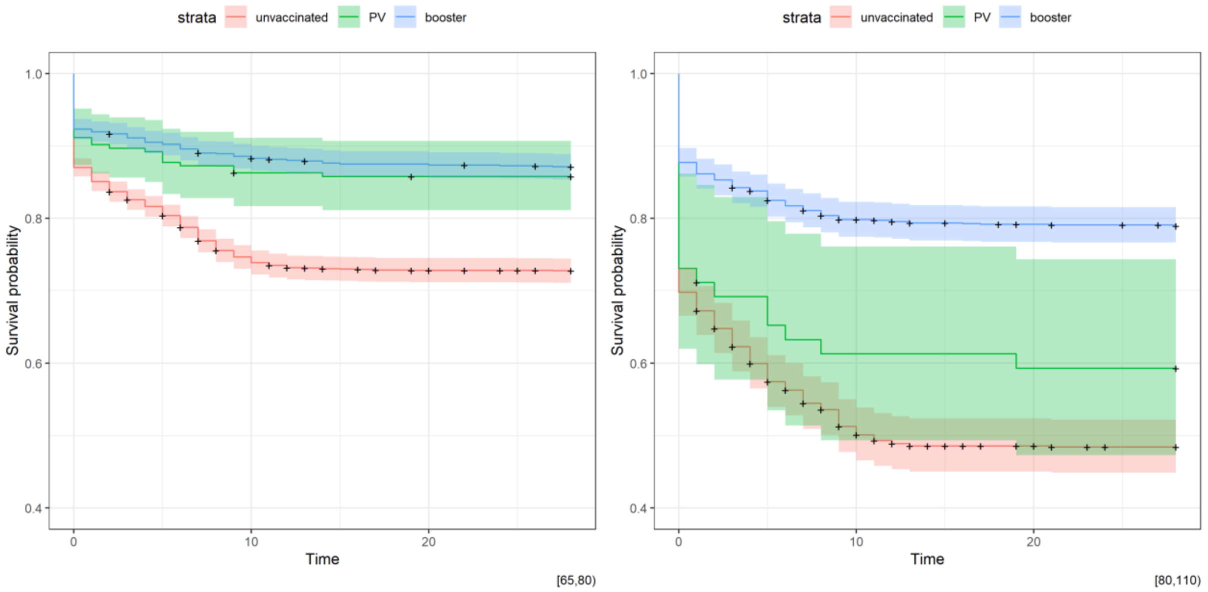

Supplementary Figure 3: Kaplan-Meier curve representing survival probability (no hospitalization linked to Covid-19 reported to the Clinical Hospital Survey) over time since PCR-confirmed symptomatic infection for a subset of the data, 12/07/2021-22/12/2021 (Delta), 65-79 years (left), 80-109 years (right), initial effect of vaccination, no prior infection (PV = primary vaccination).

The Cox-regression model is adjusted for age and time since vaccination. We present survival curves by immunity status (unvaccinated and the initial effect of primary-vaccination and booster-vaccination, all without prior infection), period of Delta-dominance (Supplementary Figure 4).

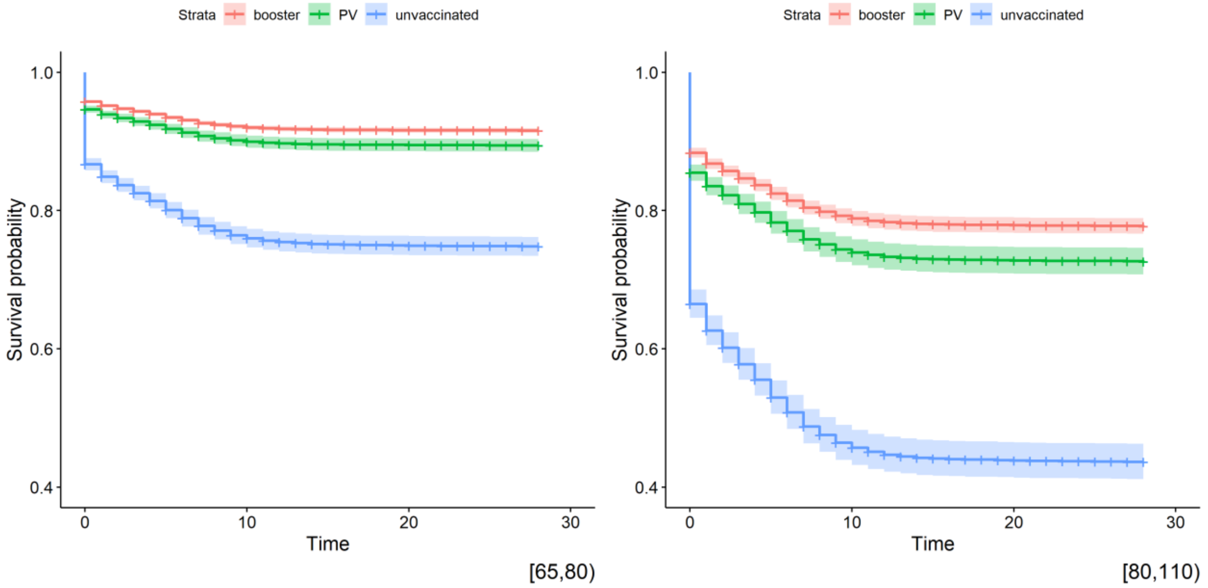

Supplementary Figure 4: Predictions from the Cox-regression model representing survival probability (no hospitalization reported to the Clinical Hospital Survey) over time since PCR-confirmed symptomatic infection , 12/07/2021-22/12/2021 (Delta), 65-79 years (left), 80-109 years (right), initial effect of vaccination, no prior infection (PV=primary vaccination).

The sample mean for time to hospitalization varies by vaccination-status and variant. For Delta: primary-vaccination 2.9 days, booster-vaccination 3.1 days and unvaccinated 3.7 days. For Omicron: booster-vaccination 1.5 days, primary-vaccination 1.9 days and unvaccinated 2 days. These estimates are not adjusted and age likely is a confounder. In addition, sample variance associated with these sample means is considerable (around 15 days)

The assumption of proportional hazards is explored by plotting the Schoenfeld-residuals over time for all variables included in the model. We used the cox.zph-function provided in the R-package survival for formal testing. We cannot reject the null-hypothesis (when setting the level of statistical significance to 0.05) that the immunity-status-variable included in the model is time-invariant. One Schoenfeld-residual plot is provided as illustration (Supplementary Figure 5). We therefore assume that the proportional hazard assumption is not violated for this variable.

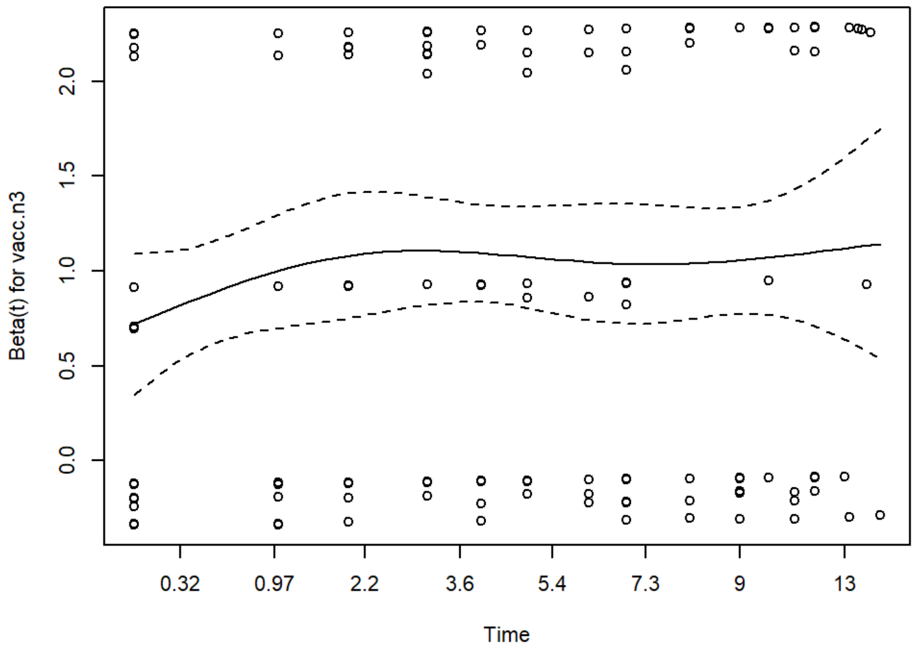

Supplementary Figure 5: Schoenfeld-residual plot to assess proportion hazards assumption, females 65 years and older, 12/07/2021-22/12/2021 (Delta), Belgium

S3. Is the VE estimated from the Clinical Hospital survey an unbiased estimator of Belgium's VE against hospitalization

For the VE against hospitalization estimated from the Clinical Hospital Survey (CHS) to be an unbiased estimator of the VE against hospitalization for Belgium, we have to assume that reporting of patients to the CHS is independent from their immunity status (vaccination status and history of prior infection). This assumption is violated if **(1)** the hospitals reporting to the CHS hospitalize patients whose immunity status is different from patients hospitalized in hospitals not reporting to the CHS and **(2)** if within the hospitals reporting to the CHS, patients with a certain immunity status are more frequently reported.

While a nationwide, exhaustive hospitalization registry for COVID-19 is unavailable in Belgium, the two assumptions can be explored through comparison with a second data source. This data source, the surge capacity survey, (SCS) contains daily, hospital-specific numbers on capacity and intakes (for COVID-19) by 'grouped' vaccination status. Vaccination info is not brand-specific, was not included prior to October 2021 and the distinction between primary-vaccination and booster-vaccination was not made until December 2021. There is no information on time since vaccination or prior infection. In addition, the information might not always be based on the vaccination-registry, but might also be self-reported. Additional information on the SCS can be obtained from Van Goethem et al. [1]. Since there is an additional correction for the province of residence in the proportional hazard analysis, the comparison can be limited to the province-level. We present a map of the Belgian provinces, total hospitalizations

during the study period and the proportion reported to the Clinical Hospital Survey (Supplementary Figure 6).

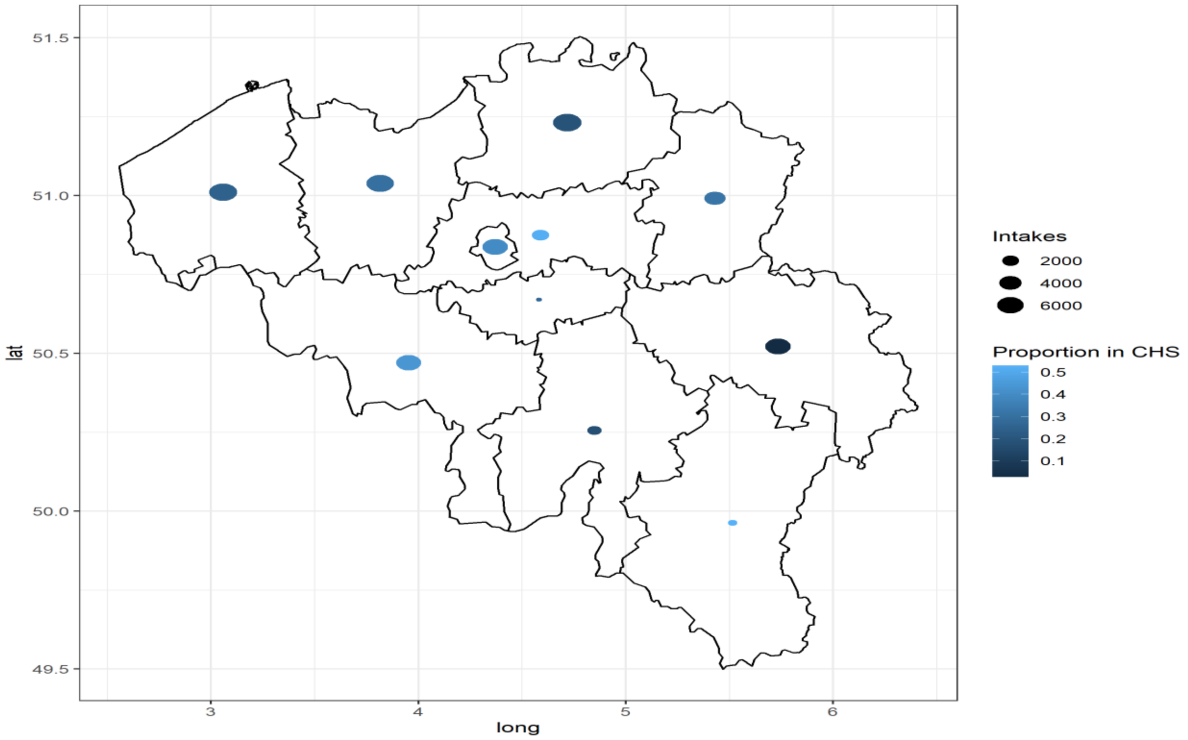

Supplementary Figure 6: Provinces of Belgium, total number of intakes over the study period as reported by the Surge Capacity Survey and their proportion reported to the Clinical Hospital Survey. 12/07/2021-26/05/2022, Belgium

We did not find the vaccination-status to differ between the Clinical Hospital Survey and the Surge Capacity Survey (Supplementary Figure 7).

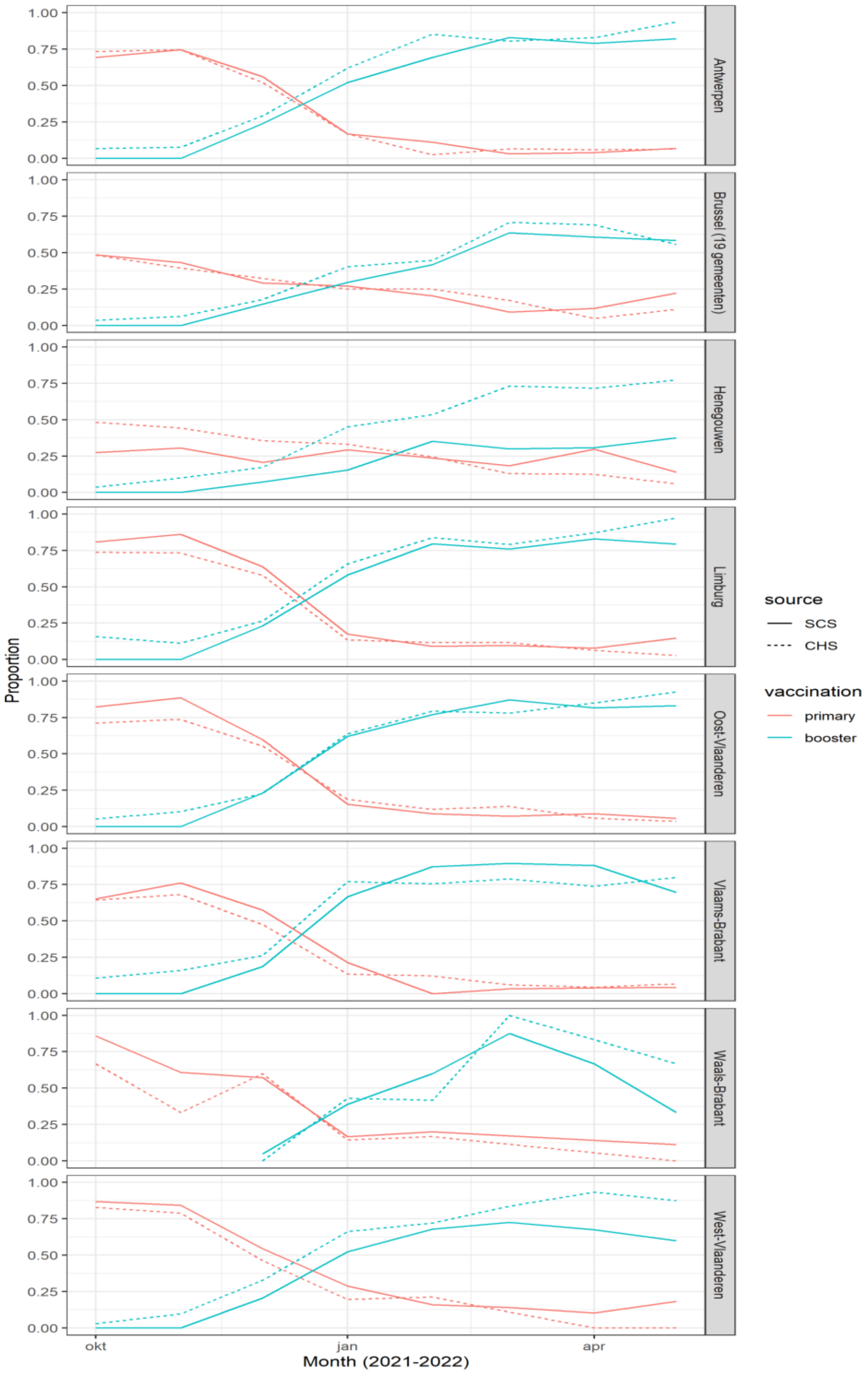

Supplementary Figure 7: New intakes by proportion vaccinated (primary-vaccination and booster-vaccination), data collected by Surge Capacity Survey (SCS) and Clinical Hospital Survey (CHS), the eight provinces with the highest reported intakes, 10/2021-05/2022, Belgium.
